# Tetracyclines Diminish *In Vitro* IFN-γ and IL-17-Producing Adaptive and Innate Immune Cells in Multiple Sclerosis

**DOI:** 10.3389/fimmu.2021.739186

**Published:** 2021-11-26

**Authors:** Despoina T. Florou, Athanasios Mavropoulos, Efthymios Dardiotis, Vana Tsimourtou, Vasileios Siokas, Athina-Maria Aloizou, Christos Liaskos, Christina Tsigalou, Christina Katsiari, Lazaros I. Sakkas, Georgios Hadjigeorgiou, Dimitrios P. Bogdanos

**Affiliations:** ^1^ Department of Rheumatology and Clinical Immunology, Faculty of Medicine, School of Health Sciences, University of Thessaly, Larissa, Greece; ^2^ Department of Neurology, Faculty of Medicine, School of Health Sciences, University of Thessaly, Larissa, Greece; ^3^ Laboratory of Microbiology, Faculty of Medicine, Democritus University of Thrace, Alexandroupolis, Greece; ^4^ Medical School, University of Cyprus, Nicosia, Cyprus

**Keywords:** multiple sclerosis, doxycycline, minocycline, proinflammatory, interferon-γ, interleukin-17, NKT cells

## Abstract

**Introduction:**

Limited data from clinical trials in multiple sclerosis (MS) reported that minocycline, a widely used antibiotic belonging to the family of tetracyclines (TCs), exerts a beneficial short-lived clinical effect A similar anti-inflammatory effect of minocycline attributed to a deviation from Th1 to Th2 immune response has been reported in experimental models of MS. Whether such an immunomodulatory mechanism is operated in the human disease remains largely unknown.

**Aim:**

To assess the *in vitro* immunomodulatory effect of tetracyclines, and in particular minocycline and doxycycline, in naïve and treated patients with MS.

**Material and Methods:**

Peripheral blood mononuclear cells from 45 individuals (35 MS patients, amongst which 15 naïve patients and 10 healthy controls, HCs) were cultured with minocycline or doxycycline and conventional stimulants (PMA/Ionomycin or IL-12/IL-18). IFN-γ and IL-17 producing T-, NK- and NKT cells were assessed by flow cytometry. The effect of TCs on cell viability and apoptosis was further assessed by flow cytometry with Annexin V staining.

**Results:**

Both tetracyclines significantly decreased, in a dose dependent manner, IFN-γ production in NKT and CD4^+^ T lymphocytes from MS patients (naïve or treated) stimulated with IL-12/IL-18 but did not decrease IFN-γ producing CD8^+^ T cells from naive MS or treated RRMS patients. They also decreased IL-17^+^ T and NKT cells following PMA and Ionomycin-stimulation. Tetracyclines did not affect the viability of cell subsets.

**Conclusion:**

Tetracyclines can *in vitro* suppress IFN-γ and IL-17- producing cells from MS patients, and this may explain their potential therapeutic effect *in vivo*.

## Introduction

Multiple Sclerosis (MS) is a chronic autoimmune inflammatory demyelinating disease of the central nervous system (CNS) (1). Based on disease course, patients can be classified into three main categories, relapsing-remitting MS (RRMS), secondary progressive MS (SPMS) and primary progressive MS (PPMS) (2). The autoimmune pathogenesis of MS is well established by translational research in patients with MS and studies based on experimental autoimmune encephalomyelitis (EAE), a model of MS (3, 4).

The activation of myelin-specific T cells followed by dysregulation of Th1, Th2 and Th17 cytokines is believed to be a major event during the initiation phase of MS (5). CD4^+^ Th1 cells produce inflammatory mediators, such as interferon-γ (IFN-γ) which lead to autoantigen-specific inflammatory attack resulting in myelin degeneration (6, 7). IL-12 and IL-18, which can synergistically induce high levels of IFN-γ expression (8), are found elevated in MS and correlate with disease activity (9, 10). In addition to Th1 cells, it is now believed that pro-inflammatory Th17 and other IFN-γ and IL-17 producing cells, such as Th17, NK and NKT cells are also significantly involved in the initiation and perpetuation of the disease (11–13). Sharp reduction of CNS-penetrating IFN-γ and IL-17 producing cells is directly linked with disease prevention or disease remission in patients with MS (14, 15).

Current therapeutic agents in MS mainly affect pro-inflammatory cytokine production (15, 16), but they lack desirable efficacy (17–19).

In addition to their well-defined antimicrobial-bacteriostatic activity, tetracyclines (TCs) (20, 21), such as minocycline and doxycycline, demonstrate neuroprotective, anti-apoptotic, anti-inflammatory and immunomodulatory properties (22–25), and have been clinically tested with encouraging results in autoimmune diseases, such as rheumatoid arthritis and MS (26–29). For example, in a recent randomized, controlled clinical trial, conversion from a clinically isolated syndrome to MS at six months was significantly lower in the minocycline compared to placebo group, but this effect was short-lived (27). Other trials failed to report an efficacy of TCs in MS (30), contradicting data from animals whereby minocycline dramatically suppresses ongoing disease activity and remarkably limits EAE disease progression (31–33). Amongst the mechanisms involved in their immunomodulatory action are inhibition on IFN-γ and TNF-α production by T-lymphocytes or monocytes (34–37). An anti-inflammatory effect of minocycline has been reported in EAE when combined with other treatments such as IFN-β, steroids, glatiramer acetate, and atorvastatin (32, 38–40). Also, Popovic et al. reported a dramatic suppression of disease activity in EAE by minocycline and a deviation of MOG-specific T cell response towards a Th2-like response (31).

The precise cellular source and mechanism of cytokine modulation by TCs in MS patients, remains largely unknown and studies conducted so far limited their assessment in particular cell-subsets, selecting either TC but not both. The aim of our study was to comparatively investigate for the first time the *in vitro* effect of both minocycline and doxycycline on IFN-γ and IL-17 production by peripheral cell populations in patients with MS (newly-diagnosed naive and treated RRMS).

## Patients and Methods

### Patients and Healthy Controls

A total of 35 patients with MS diagnosed according to the McDonald criteria (41) were included in the study, including 15 naïve (12 females, mean age of 41.4 ± 13.4 years, range 19-59) and 20 RRMS (13 females, mean age of 40.9 ± 11.4 years, range 20-61) with a mean disease duration of (11.2 ± 8.8 years, range 1-33). All patients attended the Outpatient Clinics of the Neurology Department, of the University General Hospital of Larissa, Greece. RRMS patients (9 at relapse) were on standard treatment (glatiramer acetate, n=3; natalizumab, n=6; fingolimod, n=5; fampridine, n=1; and IFN-β n=5). Blood collection of all naive MS patients was performed before initiation of corticosteroid or other drug treatment. Ten age and sex-matched healthy individuals (7 females, mean age of 37.3 ± 10.4 years) were included as healthy controls (HCs). Patients and healthy HCs had not received TCs for at least three months before blood collection.

All experimental protocols were approved by the Local Institution’s Ethical Committee of the University General Hospital of Larissa, University of Thessaly while written informed consent has been obtained from all study participants according to the declaration of Helsinki.

### PBMC Isolation and Cryopreservation

Peripheral blood samples (20-30mL) from MS patients and HCs were collected by venipuncture in preservative-free heparin tubes (50 U/mL) and aliquots were layered onto an equal volume of Ficoll-Hypaque (10ml Lymphoprep™) density gradient solution (Axis-Shield, Oslo, Norway). Peripheral blood mononuclear cells (PBMCs) were isolated by centrifugation at 300g, washed twice with RPMI-1640 (GIBCO™ -Thermo Fisher Scientific, Waltham, MA, USA), counted, and their viability, determined by trypan blue exclusion, routinely exceeded 95%. Cells were re-suspended in freezing medium containing 10% DMSO and 70% fetal calf serum (FCS), aliquoted into cryogenic vials (Corning™, Thermo Fisher Scientific), kept at −80°C for one day, and then stored in liquid nitrogen tanks until used.

### Reagents

TCs (minocycline hydrochloride and doxycycline hyclate, ≥ 98% pure) were purchased from Cayman Chemical Company, Michigan USA. Both antibiotics were reconstituted in DMSO, further aliquoted at small volumes and stored at a final concentration of 5 mg/ml. TCs were supplemented simultaneously with cell stimuli (see below) at a final concentration of 50μg/ml unless otherwise stated and remained in culture for 5 hours (see *Results* section). DMSO concentration in TC-supplemented cultures was less than 0.1%. The same DMSO concentration (0.1%) was also added to control cultures in the absence of TCs, to exclude a direct cytotoxic effect of DMSO. Recombinant IL-12 and IL-18 were purchased from R and D Systems Inc (Abington, UK) and used at a final concentration of 20 ng/ml and 25 ng/ml, respectively (8). Phorbol 12-myristate 13-acetate (PMA) and Ionomycin were obtained from Sigma-Aldrich-Merck (Gillingham, UK) and were used at 20-50 ng/ml, and 1μg/ml, respectively in order to conventionally stimulate PBMCs in a non-specific manner (42).

### Phenotypic Analysis of Peripheral Blood T and Innate Cells by Flow Cytometry

Phenotypic assessment and enumeration of peripheral blood sub-populations was performed using standard monoclonal antibodies (MoAbs) panels and protocols as described previously (43, 44). Briefly, upon thawing PBMCs were washed with serum-free RPMI-1640, counted to confirm more than 95% cell viability, pelleted, and re-suspended at 10^6^ cells/mL in RPMI culture medium supplemented with L-glutamine and 10% heat-inactivated FCS (Biosera Europe, Nuaille, France). PBMCs were seeded in 24-well plates and allowed to rest at 37°C in a CO^2^ incubator for at least one hour before stimulation. In this study we used the following mouse anti-human MoAbs: (FITC)-conjugated anti-CD3 (clone UCHT-1), (PE)- and (PE-Cy7)-conjugated anti-CD56 (clones C5.9 and B159), (PE)- and (PE-Cy7)-conjugated anti-CD4 (clone RPA-T4) and PE conjugated anti-CD8 (clone SK1). All MoAbs were obtained from BD Biosciences (Mountain View, CA, USA) and Merck-Millipore (Burlington, MA, USA). Isolated PBMCs (0.5-1 x 10^6^cells) were washed in phosphate-buffered saline (PBS) and re-suspended in staining buffer (PBS plus 1% FCS plus 0.09% sodium azide) and then incubated with labeled MoAbs specific for cell surface antigens for 30 minutes on ice and fixed with paraformaldehyde (2%). Background auto-fluorescence was monitored by equivalent 4-colour matched isotype control mouse anti-human MoAbs and formed the basis to set the cut-off for surface-positive cell discrimination. Flow cytometric analysis was performed in Guava^®^ EasyCyte8 (Merck-Millipore, Burlington, MA, USA) benchtop flow cytometer using logarithmic amplification and a forward and side scatter-based gate for total lymphocyte populations. At least 3x10^5^ events within the lymphocyte gate were collected for accurate measurement of infrequent cell subtypes.

### Flow Cytometric Analysis of Apoptotic Cells by Annexin V

IL-12 and IL-18 activated PBMCs supplemented with 50μg/ml minocycline or doxycycline were collected and stained with FITC-labeled Annexin V (BioLegend, San Diego, CA, USA), which is used to specifically target and identify apoptotic cells (45), in the presence of Annexin V binding buffer according to manufacturer’s instructions. Experiments in the absence of IL-12 and IL-18 were also performed as controls. Lymphocyte subsets were identified using fluorescent labeled mAbs directed against lymphocyte surface markers (section 2.4) and subjected to conventional FACS analysis.

### Intracellular IFN-γ Production by Peripheral Blood Cell Subsets

In order to measure intracellular IFN-γ protein production by peripheral T cells and innate NK and NKT cells, PBMCs were left untreated, or cultured in 10% RPMI supplemented with 20 ng/ml PMA plus 1μg/ml ionomycin for 5 hours in the presence of Brefeldin A (GolgiPlug™, BD Biosciences). Cells were surface stained, fixed and subsequently permeabilized using commercially available Perm/Wash buffers (BD Biosciences). Intracellular IFN-γ protein was detected using APC-conjugated MoAbs (clone 4S.B3) obtained from BD Biosciences.

### Intracellular IL-17 Production by Th17 Cells

In order to measure intracellular IL-17 protein production by peripheral Th17 cells, PBMCs were left untreated or cultured in 10% RPMI supplemented with 50 ng/ml PMA plus 1μg/ml ionomycin for 5 hours in the presence of Brefeldin A. Cells were surface stained, fixed and subsequently permeabilized using commercially available Perm/Wash buffers (BD Biosciences). Intracellular IL-17 was detected using FITC- and PE-conjugated MoAbs clones (BL-168) all obtained from BD Biosciences and Merck-Millipore.

### Statistical Analysis

Percentages of cells expressing cell surface markers and mean fluorescence intensities (MFI) were described as median of the individuals in each group. Variation in each patient group was defined by standard deviation (SD). Differences between healthy controls and patients and between patient groups one-way analysis of variance (ANOVA) and the nonparametric Mann-Whitney test. P-values smaller than 0.05 were considered significant. All graphs and statistical calculations were performed with Graph Pad Prism 9 software.

## Results

### Tetracyclines Decreased IFN-γ Producing Cells in a Dose-Dependent Manner

To assess the *in vitro* effect of either minocycline or doxycycline on IFN-γ production, antibiotics in isolation were supplemented in PBMC cell cultures at different concentrations ranging from of 0.1μg - 50μg/ml, similarly to previous reports in whole blood cultures and THP-1 human monocytes (46, 47). These concentrations are within the *in vivo* pharmacological plasma concentration levels noted following oral tetracycline administration; in human subjects who have taken oral doxycycline (200mg), doxycycline plasma concentrations (Cmax) of 1.5 to 7.0 μg/ml were usually reached within 3 h, and the drug had a half-life of 14 to 24 h (48, 49). Also, such TCs concentrations were administered in accordance with *in vivo* studies of experimental endotoxemia where doxycycline and other TCs are efficacious in downregulating inflammatory cytokines and preventing shock when the drug was injected immediately following the LPS challenge (50).

IFN-γ production was measured following stimulation with IL-12 plus IL-18 or PMA plus ionomycin for 5 hours on the basis of TCs optimal Cmax and limited half-life *in vivo*, as well as our previous published data, where both stimuli induce kinase (p38 MAPK) activation and robust IFN-γ production within 2-6hrs post stimulation in innate and adaptive cells (8).

**Supplementary Figures 1**, **2** show cumulative data from TC dose-response experiments in MS patients (n=3) and HCs (n=3). Minocycline or doxycycline decreased IFN-γ production by innate and adaptive T cells in a dose-dependent manner when IL-12 plus IL-18 was used for cell stimulation. Both drugs clearly inhibited cytokine expression in CD4^+^ T cells and NKT cells at pharmacological concentrations (1μg/ml-10μg/ml). However, the maximal inhibitory effect was noted when IL-12 plus IL-18 and 50μg/ml minocycline or doxycycline were used (see below). In all tested concentrations there was no detrimental effect of TCs on cell viability, as assessed by microscopic evaluation and trypan blue exclusion in line with previously published observations (47). The effect of TCs on cell viability was further assessed using Annexin V staining. As shown in **Supplementary Figures 3**, **4** neither antibiotic exerted a toxicity effect influencing the viability, apoptosis and percentages of any cell subset at the maximal concentration (50μg/ml). Neither minocycline nor doxycycline exerted an inhibitory effect on IFN-γ producing CD3^+^ and non CD3^+^ cell subsets from naive MS and RRMS patients, after stimulation with PMA and Ionomycin (**Supplementary Figures 2**, **6**). There was a marginal statistically significant decrease in IFN-γ^+^ NKT (CD3^+^CD56^+^) cells following treatment with doxycycline (p = 0.04, n = 9) (**Supplementary Figure 7**). Thus, all subsequent experiments were performed using IL-12 plus IL-18 and minocycline or doxycycline at 50μg/ml.

### Tetracyclines Decreased IFN-γ Producing CD4^+^ T Cells in MS

**Figure 1:**
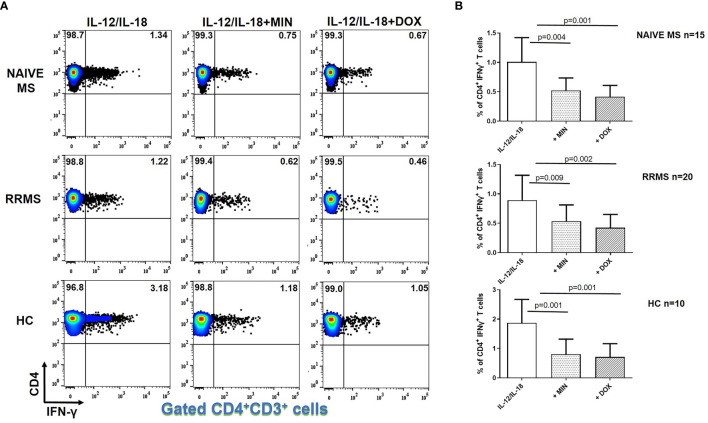
Tetracycline-mediated inhibition of CD4^+^ IFNγ^+^ T cells. PBMCs from naïve MS patients (n=15), RRMS patients (n=20) and HCs (n=10) were seeded in cell culture plates (1 x 10^6^ per well) and stimulated with IL-12 plus IL-18 (IL-12/IL-18), IL-12/IL-18 plus minocycline (MIN) and IL-12/IL-18 plus doxycycline (DOX) for 5h. Cells were collected, washed, surface stained with appropriate monoclonal antibodies an analyzed for intracellular IFN-γ production by flow cytometry (see also methods section). Individual cell subsets were sub-gated according to expression of CD3 and CD4 surface epitopes. **(A)** Flow cytometry dot-plots showing the frequency of CD4^+^IFNγ^+^ T cells in IL-12/IL-18, IL-12/IL-18/MIN and IL-12/IL-18/DOX treated cells from representative MS cases and HCs. **(B)** Box and whiskers graphical representation showing significant reduction in the percentages of IFN-γ-producing CD4^+^ T cells in the presence of tetracyclines in naïve MS, RRMS and HCs.

The flow cytometric gating strategy followed for phenotypic analysis of different cell subsets, including CD4^+^ T cells, is shown at **Supplementary Figures 3**, **5**. In general, we observed a statistically significant reduction in IFN-γ producing CD4^+^ T cells from both MS patients and HCs in the presence of minocycline or doxycycline. **Figure 1** illustrates representative cases and cumulative data. The percentage of IFN-γ^+^ CD4^+^ T lymphocytes following IL-12 plus IL-18 stimulation in naïve MS patients (n=15) decreased from 1.1 ± 0.41% to 0.53 ± 0.21% in the presence of minocycline (p = 0.004) and to 0.42 ± 0.20% in the presence of doxycycline (p = 0.001). The percentage of IFN-γ^+^ CD4^+^ T cells in RRMS patients (n=20) decreased from 0.89 ± 0.43% to 0.53 ± 0.27% in the presence of minocycline (p = 0.009) and to 0.44 ± 0.22% in the presence of doxycycline (p = 0.002). In HCs (n=10), the percentage of IFN-γ^+^ CD4^+^ T lymphocytes was reduced from 1.87 ± 0.79% to 0.80 ± 0.51% in the presence of minocycline (p = 0.0015) and to 0.71 ± 0.45% in the presence of doxycycline (p = 0.001) (**Figure 1**).

**Figure 2:**
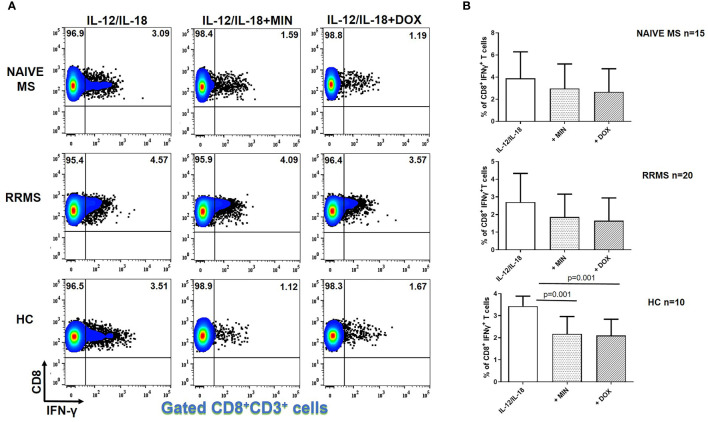
Tetracycline-mediated inhibition of CD8^+^ IFNγ^+^ T cells. PBMCs from naïve MS patients (n=15), RRMS patients (n=20) and HCs (n=10) were seeded in cell culture plates (1 x 10^6^ per well) and stimulated with IL-12 plus IL-18 (IL-12/IL-18), IL-12/IL-18 plus minocycline (MIN) and IL-12/IL-18 plus doxycycline (DOX) for 5h. Cells were collected, washed, surface stained with appropriate monoclonal antibodies an analyzed for intracellular IFN-γ production by flow cytometry (see also methods section). Individual cell subsets were sub-gated according to expression of CD3 and CD8 surface epitopes. **(A)** Flow cytometry dot-plots showing the frequency of CD8^+^IFNγ^+^ T cells in IL-12/IL-18, IL-12/IL-18/MIN and IL-12/IL-18/DOX treated cells from representative MS cases and HCs. **(B)** Box and whiskers graphical representation showing significant reduction in the percentages of IFNγ-producing CD8^+^ T cells in the presence of tetracyclines in HCs but not naïve MS and RRMS patients.

Minocycline and doxycycline did not decrease IFN-γ producing CD8^+^ T cells from naive MS (n=15) or RRMS patients (n=20) (**Figure 2**). However, in HCs (n=10), the percentage of IFN-γ^+^CD8^+^ T lymphocytes was reduced from 3.43 ± 0.45% to 2.18 ± 0.78% in the presence of minocycline (p = 0.001) and to 2.10 ± 0.75% following doxycycline supplementation (p = 0.001) (**Figure 2**).

### Tetracyclines Decreased IFN-γ Producing NKT Cells in MS

**Figure 3:**
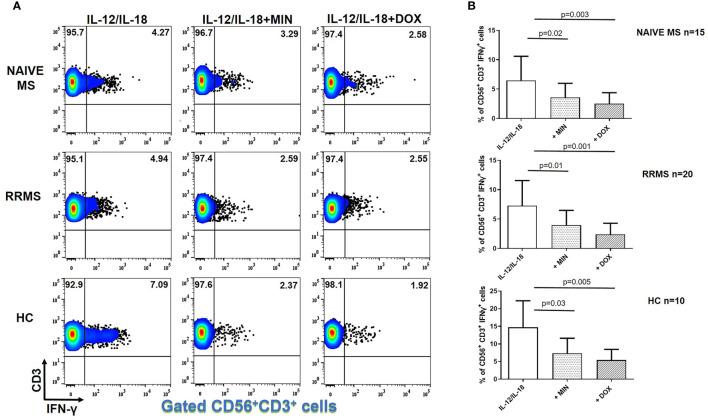
Tetracycline-mediated inhibition of CD56^+^CD3^+^IFN-γ^+^ T cells. PBMCs from naïve MS patients (n=15), RRMS patients (n=20) and HCs (n=10) were seeded in cell culture plates (1 x 10^6^ per well) and stimulated with IL-12 plus IL-18 (IL-12/IL-18), IL-12/IL-18 plus minocycline (MIN) and IL-12/IL-18 plus doxycycline (DOX) for 5h. Cells were collected, washed, surface stained with appropriate monoclonal antibodies an analyzed for intracellular IFN-γ production by flow cytometry (see also methods section). Individual cell subsets were sub-gated according to expression of CD3 and CD56 surface epitopes. **(A)** Flow cytometry dot-plots showing the frequency of IFN-γ^+^ NKT cells (gated CD56^+^CD3^+^ cells two-dimensionally depicted in CD3 *vs* IFN-γ plots) in IL-12/IL-18, IL-12/IL-18/MIN and IL-12/IL-18/DOX treated cells from representative MS cases. **(B)** Box and whiskers graphical representation showing significant reduction in the percentages of IFN-γ-producing NKT cells in the presence of tetracyclines in naïve MS, RRMS and HCs.

IFN-γ^+^ NKT lymphocytes following IL-12 plus IL-18 stimulation in naïve MS (n=15) patients decreased from 6.49 ± 4.10% to 3.61 ± 2.39% in the presence of minocycline (p = 0.02) and to 2.52 ± 1.87% in the presence of doxycycline (p = 0.003) (**Figure 3**). The percentage of IFN-γ^+^ NKT cells in RRMS patients (n=20) decreased from 7.34 ± 4.40% to 3.92 ± 2.51% in the presence of minocycline (p = 0.01) and to 2.40 ± 1.89% in the presence of doxycycline (p = 0.001). In HCs (n=10), the percentage of IFN-γ^+^ NKT lymphocytes was reduced from 14.73 ± 7.53% to 7.35 ± 4.27% following minocycline supplementation (p = 0.03) and to 5.41 ± 3.07% following doxycycline supplementation (p = 0.005).

### Tetracyclines Did Not Affect IFN-γ Producing NK Cells in MS

**Figure 4:**
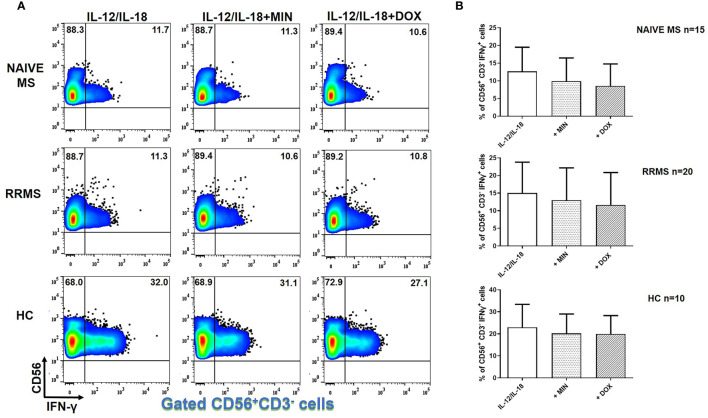
Tetracyclines have no significant effect on IFN-γ^+^ NK cells. PBMCs from naïve MS patients (n=15), RRMS patients (n=20) and HCs (n=10) were seeded in cell culture plates (1 x 10^6^ per well) and stimulated with IL-12 plus IL-18 (IL-12/IL-18), IL-12/IL-18 plus minocycline (MIN) and IL-12/IL-18 plus doxycycline (DOX) for 5h. Cells were collected, washed, surface stained with appropriate monoclonal antibodies an analyzed for intracellular IFN-γ production by flow cytometry (see also methods section). Individual cell subsets were sub-gated according to expression of CD3 and CD56 surface epitopes. **(A)** Flow cytometry dot-plots showing the frequency of CD56^+^CD3^-^IFNγ^+^ NK cells (gated CD56^+^CD3^-^ cells two-dimensionally depicted in CD56 *vs* IFN-γ plots) in IL-12/IL-18, IL-12/IL-18/MIN and IL-12/IL-18/DOX treated cells from representative MS cases. **(B)** Box and whiskers graphical representation show no significant reduction in the percentages of IFN-γ-producing NK cells in the presence of tetracyclines in either HCs or MS patients.

In IL-12 and IL-18- stimulated NK cells, there was no difference in IFN-γ^+^ cells in the presence of minocycline or doxycycline in naïve MS (n=15), RRMS (n=20) or HCs (n=10) (**Figure 4**).

### Tetracyclines Decreased IL-17A Producing CD4^+^ T Cell and NKT Cells in MS

**Figure 5:**
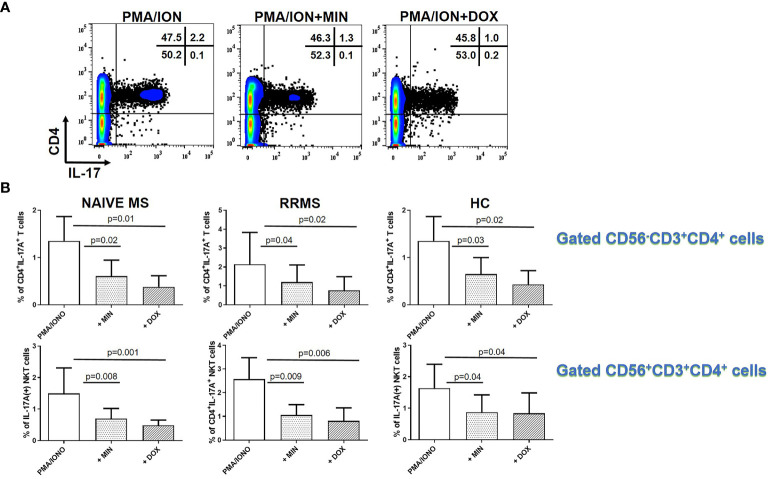
Tetracycline-mediated inhibition of CD4^+^CD3^+^IL-17A^+^ T cells and CD56^+^CD4^+^IL-17A^+^ T (NKT) cells. PBMCs from naïve MS patients (n=9), RRMS patients (n=9) and HCs (n=9) were seeded in cell culture plates (1 x 10^6^ per well) and stimulated with PMA plus ionomycin (PMA/ION), PMA/ION plus minocycline (MIN) and PMA/ION plus doxycycline (DOX) for 5h. Cells were collected, washed, surface stained with appropriate monoclonal antibodies an analyzed for intracellular IL-17A production by flow cytometry (see also methods section). **(A)** Flow cytometry dot-plots showing the frequency of total CD4^+^IL-17A^+^ cells in PMA/IONO, PMA/IONO/MIN and PMA/IONO/DOX treated cells from a representative naïve MS case. **(B)** CD4^+^IL-17A^+^ cells were sub-divided according to expression of CD3 or CD56 surface epitopes. Box and whiskers graphical representation showing significant reduction in the percentages of IL-17A-producing T and NKT cells in the presence of tetracyclines in naïve MS patients, RRMS patients and HCs.

We also investigated the effect of TCs on IL-17A production in PMA plus ionomycin activated PBMCs from patients with naïve MS (n=9), RRMS patients (n=9) and HCs (n=9). As shown in **Figure 5**, TCs decreased IL-17 production from CD4-expressing cells. These consisted of CD3^+^CD4^+^ T cells and CD56^+^CD4^+^ NKT cells. In patients with naïve MS the percentage of CD4^+^IL-17A^+^ T cells decreased from 1.77 ± 1.33% to 0.74 ± 0.43% in the presence of minocycline (p = 0.02) and to 0.55 ± 0.44% in the presence of doxycycline (p = 0.01) (**Figure 5**). In RRMS patients the percentage of CD4^+^IL-17A^+^ T cells decreased from 2.14 ± 1.68% to 1.2 ± 0.9% in the presence of minocycline (p = 0.04) and to 0.76 ± 0.71% in the presence of doxycycline (p = 0.02). In HCs the percentage of CD4+IL-17A+ T cells decreased from 1.35 ± 0.51% to 0.64 ± 0.35% in the presence of minocycline (p = 0.01) and to 0.43 ± 0.29% in the presence of doxycycline (p = 0.02).

The percentage of CD4^+^IL-17A^+^ NKT lymphocytes in naïve MS patients (n=9) decreased from 1.49 ± 0.80% to 0.69 ± 0.33% in the presence of minocycline (p = 0.008) and to 0.48 ± 0.17% in the presence of doxycycline (p = 0.001). The percentage of CD4^+^IL-17A^+^ NKT lymphocytes in RRMS patients (n=9) decreased from 2.59 ± 0.90% to 1.09 ± 0.43% in the presence of minocycline (p = 0.009) and to 0.8 ± 0.55% in the presence of doxycycline (p = 0.006). The percentage of CD4^+^IL-17A^+^ NKT lymphocytes in HCs (n=9) decreased from 1.63 ± 0.75% to 0.87 ± 0.54% in the presence of minocycline (p = 0.04) and to 0.84 ± 0.64% in the presence of doxycycline (p = 0.04).

## Discussion

The major finding of our study is that of an inhibitory effect of minocycline and doxycycline on IFN-γ and IL-17 producing cells in a cell subset-related, dose-dependent manner in patients with MS, with the most prominent effect being noted in CD4^+^ T and NKT lymphocytes.

Our results are of importance for studies conducted in humans, as previous studies in MS have only been limited to EAE, with their findings emphasizing an anti-inflammatory role of antibiotics and in particular minocycline, in synergy with prednisone (51), IFN-β1 (52) glatiramer acetate (38) or atorvastatin (39). While studies in EAE showed that minocycline attenuates the disease by reducing T cell infiltration into the spinal cord without affecting the cytokine production profile (33), our human data clearly demonstrate significant reduction of IFN-γ (and/or IL-17) by both T cells and NK/NKT cells, the inhibition varying amongst different cell types. No clear data on the effect of doxycycline on pro-inflammatory cytokine production in EAE currently exist, though our human data support the notion that this antibiotic also diminishes the ability of adaptive and innate immunity cells to produce IFN-γ and IL-17. In support, doxycycline decreased inflammatory infiltration of T-cell, B-cell and macrophage infiltration, sharply diminished IL-17 production and attenuated demyelination in sciatic nerves of rats with experimental autoimmune neuritis, a model of human inflammatory demyelinating polyneuropathies (53).

In our cohort of patients with MS, a comparable TC-mediated reduction of cytokine production was noted between naïve and treated RRMS patients. Subsequent stratified analysis amongst patients receiving different therapies revealed no statistically significant differences regarding the levels of TC-mediated cytokine inhibition within CD4^+^ T and NKT cells. It remains to be seen whether the *in vitro* effect of TC could be augmented following supplementation of MS-related immunomodulatory agents and whether this effect could be seen *in vivo*.

Clinical trials using minocycline, alone or in combination with conventional therapeutic agents, have produced inconclusive results, some demonstrating beneficial effect on the risk of conversion from a clinically isolated syndrome to MS (27), while others failed to show a superiority of minocycline when added to IFN β-1a (30).

Those studies by no means can de-emphasize the importance of our *in vitro* study. In rheumatoid arthritis, a typical autoimmune disease, both experimental and human data reveal a beneficial effect of TCs in combination with methotrexate over methotrexate alone, especially in early disease state, suggesting that timing of initiation of TCs may be of importance (54, 55). This effect is clearly attributed to the dose-dependent inhibition of T cell proliferation and reduction of IFN-γ and other pro-inflammatory cytokines (56), a finding that perfectly fits with those obtained in our study.

The inhibitory effect of antibiotics on IFN-γ was noted after the physiological stimulation of PBMC with IL-12 and IL-18 but not with PMA and ionomycin, a phenomenon, which requires further discussion. Previous work has shown that the effect of minocycline on cytokine production by T-cells and monocytes is stimulus specific, as T cells stimulated by a Ca^2^+-independent manner exhibited a decrease in TNF-alpha mRNA in the presence of minocycline, whereas the TNF-alpha mRNA level remained unaffected by minocycline when cells were stimulated in a Ca^2^+-dependent manner, like in the case of PMA/ionomycin stimulation (36, 56). The limitation of the *in vitro* studies, even if those are conducted in patients with MS and not in animal models of MS, cannot be ignored. While no *in vivo* studies have looked at the effect of TCs in IFN-γ or IL-17 producing T and NK cell-subsets in patients with MS, a study in EAE has provided data of interest. An early report by Popovic et al. (31) found that minocycline administration suppressed migration of inflammatory cells into CNS and further activation by a direct effect on the cytokine milieu in EAE. Treatment with minocycline shifted the balance from Th-1 to Th-2 and resulted in enhanced IL-10, reduced TNF-α and a minimal effect on IFN-γ production, as measured by ELISA in cell-culture supernatants (31). The investigation of cell-subset T cell specific cytokine production was not included in the aims of that study. Those latter data further emphasize the need for a vigorous attempt to assess the *in vivo* the effect of TCs on T-cell activation and cell-subset specific pro- and anti-inflammatory cytokine production in patients with MS in well-designed clinical trials.

## Conclusion

In conclusion, this is the first *in vitro* study to show that minocycline and doxycycline have a significant inhibitory effect in CD4^+^ T and NKT cell producing IFN-γ and/or IL-17 in patients with MS (naïve or under treatment). If this is proved *in vivo* in prospectively collected biological material from MS patients under treatment with these tetracyclines, it may have potential clinical relevance. *In vivo* inhibition of IFN-γ (and possibly IL-17)-producing NKT cells, for example, is a favorable pre-requisite for successful remission in patients with MS (57–59), and the effect of tetracyclines towards achieving this goal may provide an additional therapeutic tool, most likely in combination with standard treatment regimen in stratified cohorts of patients.

## Data Availability Statement

The raw data supporting the conclusions of this article will be made available by the authors, without undue reservation.

## Ethics Statement

The studies involving human participants were reviewed and approved by Ethics and Scientific Committee, University General Hospital of Larissa, University of Thessaly, Faculty of Medicine, School of Health Sciences. The patients/participants provided their written informed consent to participate in this study.

## Author Contributions

DF, AM, and ED contributed equally. LS, GH, and DB had the original idea. AM, ED, LS, GH and DB scripted considerable part of original draft. DF, AM, ED, CK, LS, and DB had performed extensive literature search and scripted relevant parts of the manuscript. DF, ED, VT, AA, VS, CT, and GH collected clinical and biological material and clinical data. DF, AM and CL analyzed the data. DF and DB prepared the artwork. DB, DF, and AM had edited the final version of the manuscript. DB and ED provided overall supervision. All authors performed extensive literature search and approved the final version of the manuscript. All authors contributed to the article and approved the submitted version.

## Funding

This research was funded in part by the Special Account for Research Grants University of Thessaly, grant number 6357, 5158 and 5847.

## Conflict of Interest

Author ED: Allergan, Novartis, Genesis, ELPEN, Bayer, Teva, Merck- Serono, Genzyme-Sanofi, Roche, UCB - speaker or chairman horonaria, advisory or travel grants, and clinical research-educational support grants. Author DB: AbbVie, Novartis, Genesis, ELPEN, Pfizer, Aenorasis, Menarini, Kopper, ITF Hellas, Roche, MSD, GSK, Hospital Line - speaker or chairman horonaria or paid investigator or clinical research and educational support grants.

The remaining authors declare that the research was conducted in the absence of any commercial or financial relationships that could be construed as a potential conflict of interest.

## Publisher’s Note

All claims expressed in this article are solely those of the authors and do not necessarily represent those of their affiliated organizations, or those of the publisher, the editors and the reviewers. Any product that may be evaluated in this article, or claim that may be made by its manufacturer, is not guaranteed or endorsed by the publisher.

## Supplementary Material

Supplementary Figure 1 IFN-γ producing PBMC subsets following stimulation with IL-12 plus IL-18 in the presence of different doses of tetracyclines. The *in vitro* effect of either minocycline or doxycycline supplemented at different concentrations ranging from of 0.1μg - 50μg/ml on IFN-γ production was assessed in PBMC cell cultures of MS patients (n=3) and HCs (n=3) following stimulation with IL-12 plus IL-18 for 5 hours (Box and whiskers graphical representation of cumulative data with standard deviation errors bars are shown). *P < 0.05 **P < 0.005 ***P < 0.0005.

Supplementary Figure 2 IFN-γ producing PBMC subsets following stimulation with PMA plus ionomycin in the presence of different doses of tetracyclines. The *in vitro* effect of either minocycline or doxycycline supplemented at different concentrations ranging from of 0.1μg - 50μg/ml on IFN-γ production was assessed in PBMC cell cultures of MS patients (n=3) and HCs (n=3) following stimulation with PMA plus ionomycin for 5 hours. (Box and whiskers graphical representation of cumulative data with standard deviation errors bars are shown). Data show a dose-depending inhibition only in IFN-γ producing NKT cells *P < 0.05 (see also **Supplementary Figure 7**).

Supplementary Figure 3 Tetracyclines do not affect PBMC flow cytometric forward/side scatter characteristics and proportions of sub-gated cell subsets. Representative flow cytometric assessment (MS case) of the *in vitro* effect of either minocycline or doxycycline supplemented at the highest concentration (50μg/ml) on PBMC cell viability and on cytometric analysis characteristics based on size/granularity (FSC/SSC) and phenotypic discrimination of the proportions of individual cell subset.

Supplementary Figure 4 Tetracyclines do not induce apoptosis of cell subsets. Representative flow cytometric illustration (MS case) of the *in vitro* effect of either minocycline or doxycycline supplemented at the highest concentration (50μg/ml) on apoptosis of sub-gated cell subpopulations of CD4 and CD8 T cells assessed by annexin V staining. Data are suggestive of lack of an *in vitro* effect of tetracyclines in inducting apoptosis and affecting viability.

Supplementary Figure 5 Representative flow cytometric gating strategy of tetracycline treated IL-12 plus IL-18 stimulated PBMC subsets. Individual cell subsets from a representative MS case were sub-gated according to expression of CD3, CD4, CD8 and CD56 surface markers. All surface epitopes were sufficiently maintained and detected following cell activation with IL-12 plus IL-18 in the presence of minocycline and doxycycline.

Supplementary Figure 6 Flow cytometric illustration of tetracycline-mediated effects on IFNγ production by PMA/ionomycin and IL-12 plus IL-18 stimulated CD3^+^ and non-CD3^+^ cell subsets from MS patients. Data of the effect of tetracyclines using different stimuli in a representative naïve MS case are shown in A and B and in a representative RRMS are shown in C and D. PBMCs were analyzed for IFN-γ production by flow cytometry following minocycline (MIN) or doxycycline (DOX) supplementation (50μg/ml) and simultaneous treatment with PMA plus ionomycin **(A, C)** or IL-12 plus IL-18 stimulation **(B, D)**. CD3^+^ and non-CD3^+^ cell subsets were sub-gated according to expression of CD3 surface epitope.

Supplementary Figure 7 Doxycycline-mediated inhibition of CD56^+^CD3^+^IFNγ^+^ T cells subsets following stimulation with PMA plus ionomycin. PBMCs from naïve MS patients (n=9) were analyzed for IFN-γ production by flow cytometry following minocycline (MIN) or doxycycline (DOX) supplementation and simultaneous PMA plus ionomycin stimulation (see also methods section). NKT cells were sub-gated according to expression of CD3 and CD56 surface markers. **(A)** CD3 versus IFN-γ Flow cytometry dot-plots in sub-gated NKT cells showing the frequency of IFN-γ^+^ NKTs following PMA/IONO, PMA/IONO/MIN and PMA/IONO/DOX treatment. **(B)** Box and whiskers graphical representation showing significant reduction in the percentages of IFN-γ-producing NKT cells in the presence of doxycycline in naïve MS.

Supplementary Figure 8 CD4^+^IFN-γ^+^ cells represent T helper 1 (TH1) cells and not other CD4^+^ subsets like macrophage or DC subsets. PBMCs from MS patients were analyzed for IFN-γ production by flow cytometry following minocycline (MIN) or doxycycline (DOX) supplementation and IL-12 plus IL-18 stimulation (see also methods section).Th1 cells were assessed by staining against CD3, CD4 and intracellular IFN-γ simultaneously. A representative case of n=5 is illustrated where the effect of minocycline and doxycycline on IFN-γ production is clearly seen on CD3^+^ and CD4^+^ ie Th1 cells.

## Abbreviations

CNS, Central Nervous System; EAE, Experimental Autoimmune encephalomyelitis; FCS, Fetal Calf Serum; MoAb, Monoclonal Antibody; MS, Multiple Sclerosis; PMA, Phorbol Myristate Acetate; TC, Tetracycline.
