# Supplementary figures and images for: Tetracyclines Diminish In Vitro IFN-γ and IL-17-Producing Adaptive and Innate Immune Cells in Multiple Sclerosis
Source: Front Immunol. 2021 Nov 26;12:739186. doi: 10.3389/fimmu.2021.739186 (PMC8662812; doi:10.3389/fimmu.2021.739186)

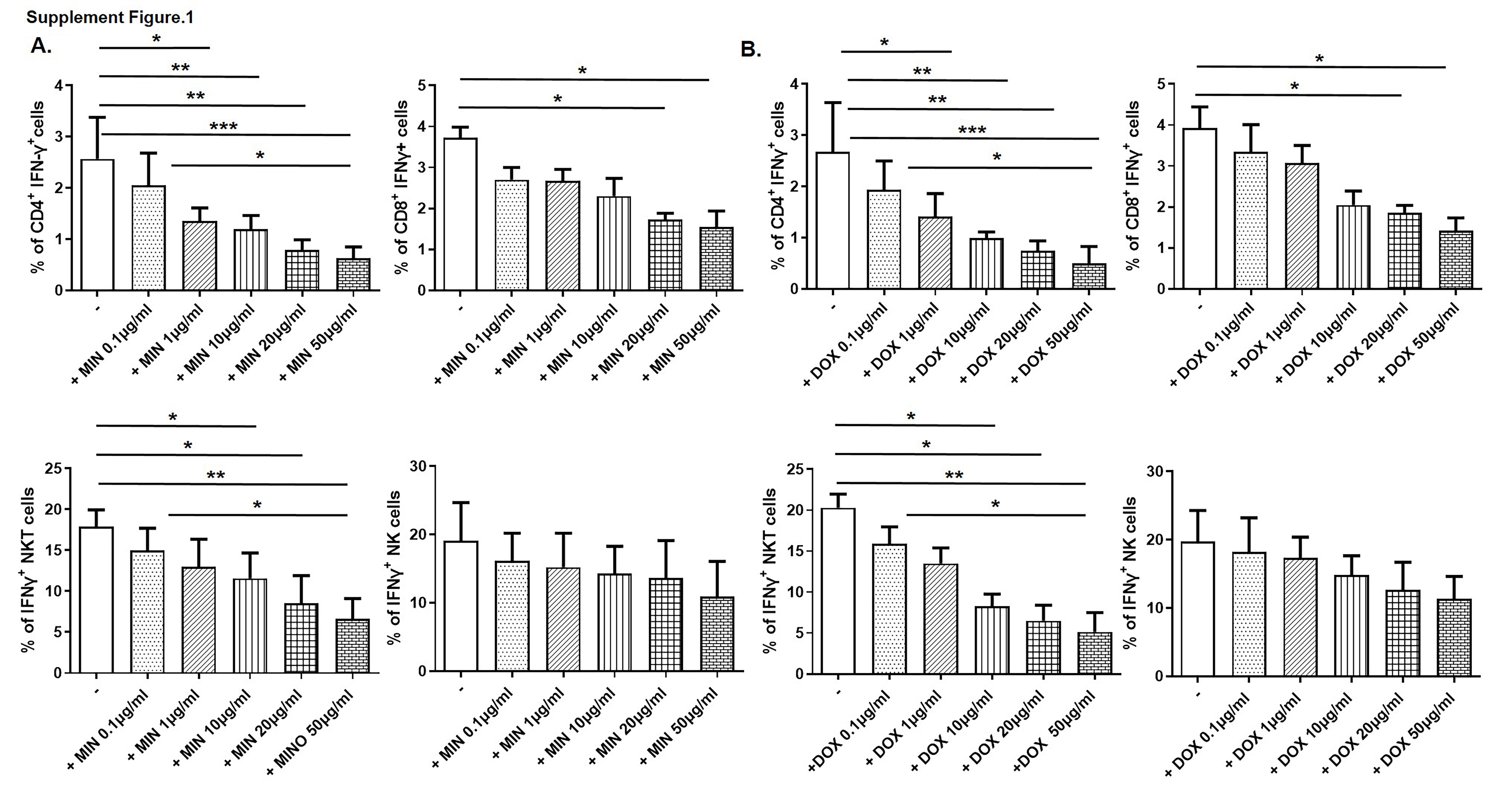

Supplement: Supplementary Figure 1 — IFN-γ producing PBMC subsets following stimulation with IL-12 plus IL-18 in the presence of different doses of tetracyclines. The in vitro effect of either minocycline or doxycycline supplemented at different concentrations ranging from of 0.1μg - 50μg/ml on IFN-γ production was assessed in PBMC cell cultures of MS patients (n=3) and HCs (n=3) following stimulation with IL-12 plus IL-18 for 5 hours (Box and whiskers graphical representation of cumulative data with standard deviation errors bars are shown). *P < 0.05 **P < 0.005 ***P < 0.0005. [file Image_1.jpeg]

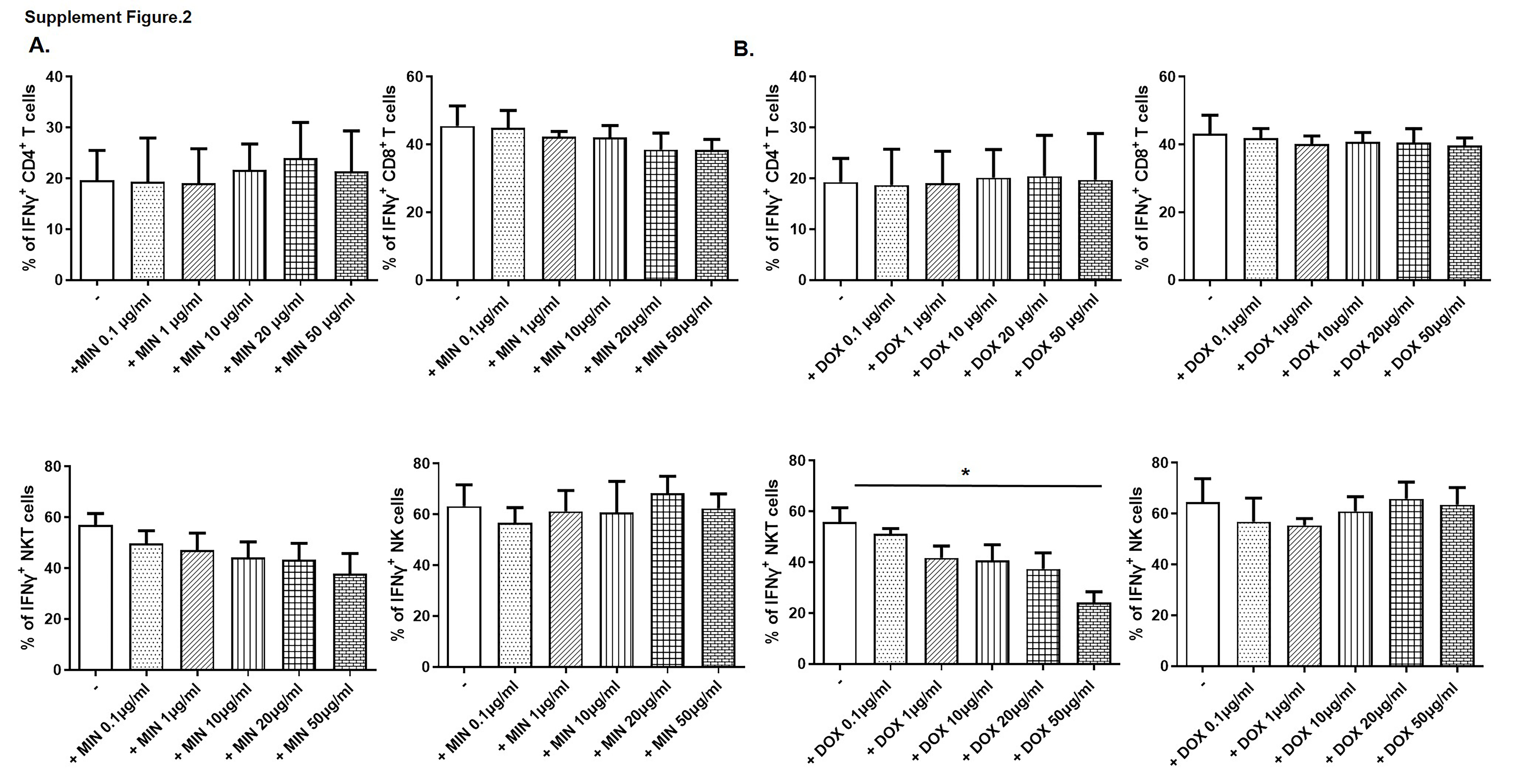

Supplement: Supplementary Figure 2 — IFN-γ producing PBMC subsets following stimulation with PMA plus ionomycin in the presence of different doses of tetracyclines. The in vitro effect of either minocycline or doxycycline supplemented at different concentrations ranging from of 0.1μg - 50μg/ml on IFN-γ production was assessed in PBMC cell cultures of MS patients (n=3) and HCs (n=3) following stimulation with PMA plus ionomycin for 5 hours. (Box and whiskers graphical representation of cumulative data with standard deviation errors bars are shown). Data show a dose-depending inhibition only in IFN-γ producing NKT cells *P < 0.05 (see also Supplementary Figure 7 ). [file Image_2.jpeg]

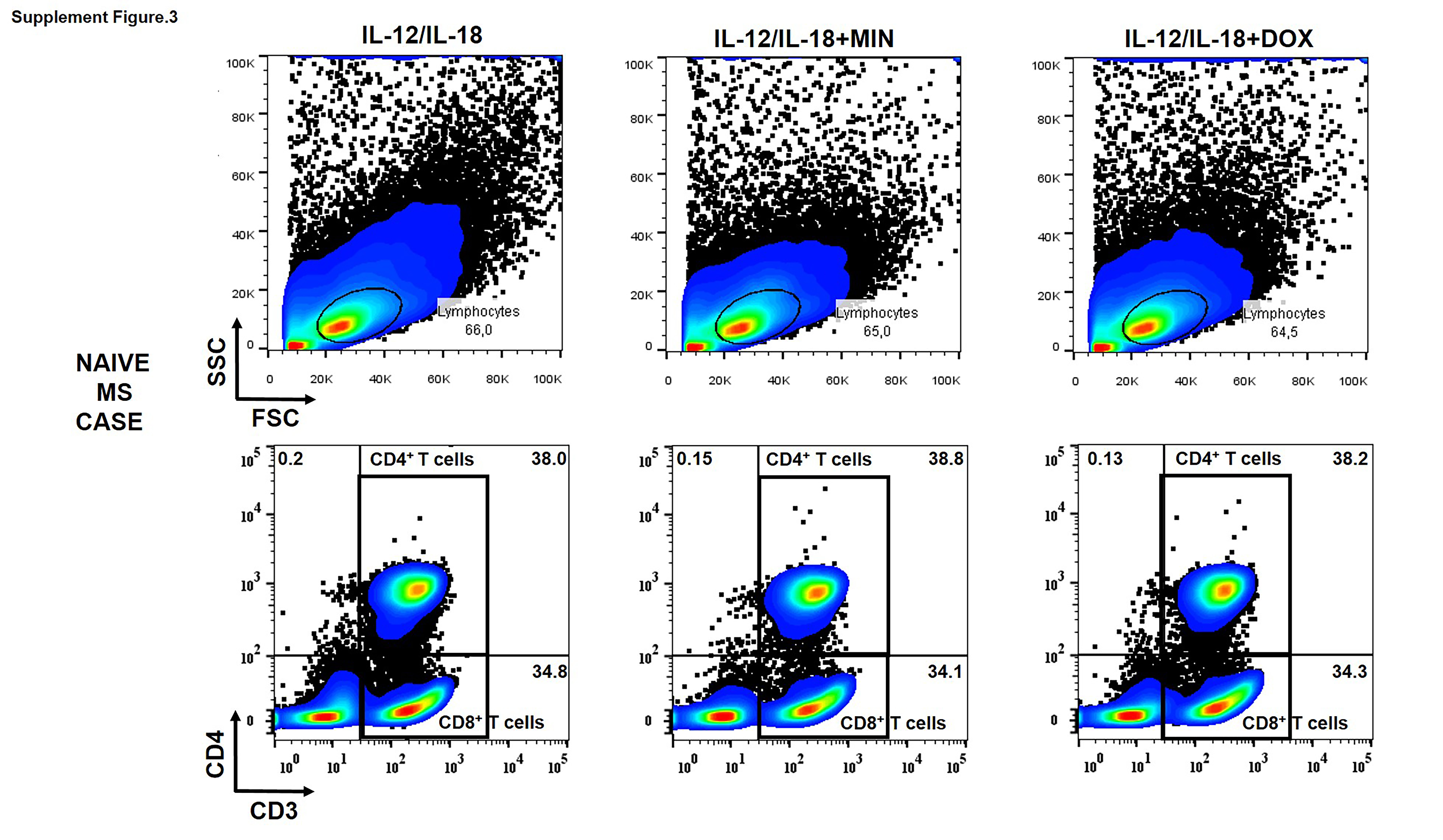

Supplement: Supplementary Figure 3 — Tetracyclines do not affect PBMC flow cytometric forward/side scatter characteristics and proportions of sub-gated cell subsets. Representative flow cytometric assessment (MS case) of the in vitro effect of either minocycline or doxycycline supplemented at the highest concentration (50μg/ml) on PBMC cell viability and on cytometric analysis characteristics based on size/granularity (FSC/SSC) and phenotypic discrimination of the proportions of individual cell subset. [file Image_3.jpeg]

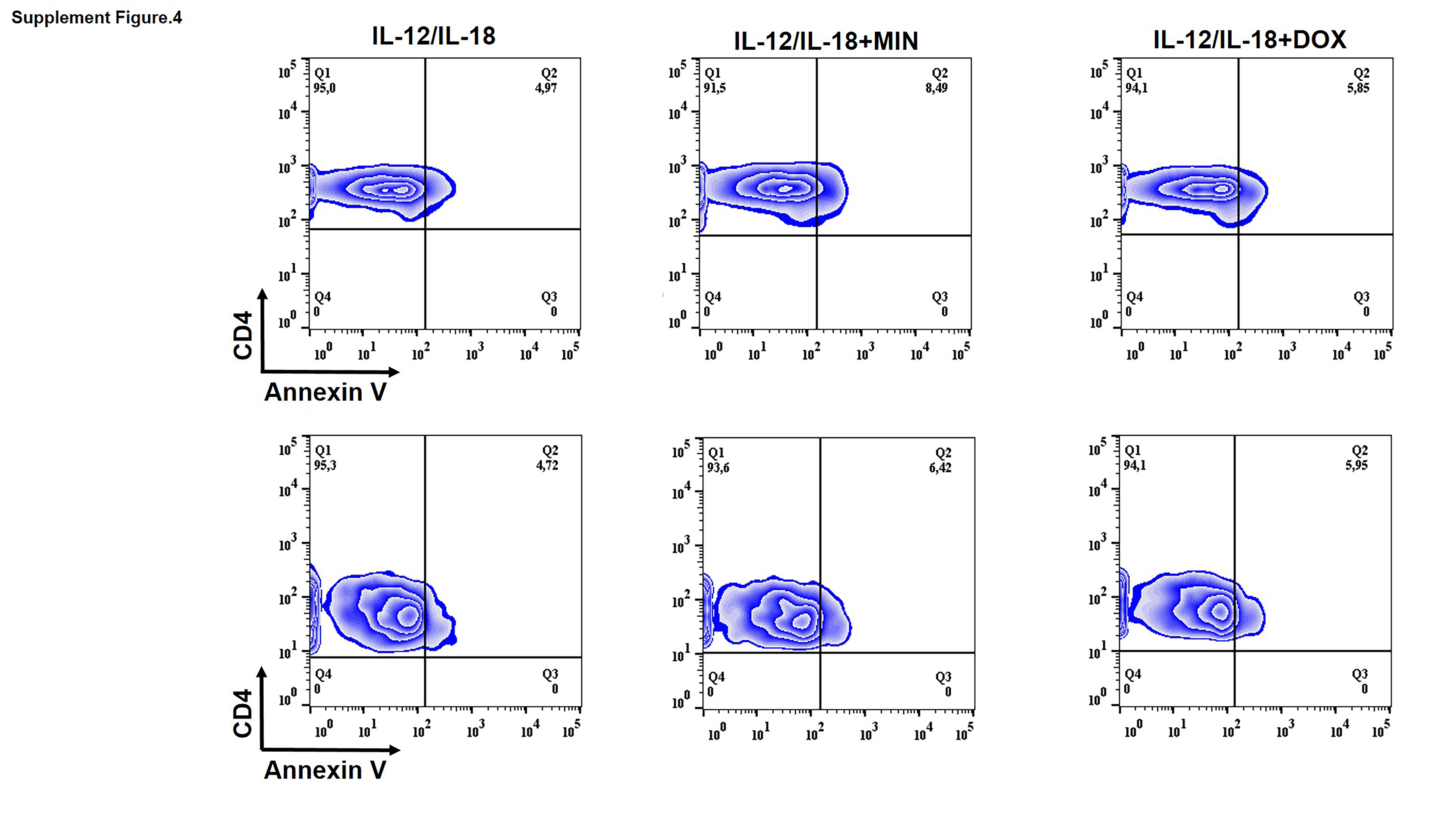

Supplement: Supplementary Figure 4 — Tetracyclines do not induce apoptosis of cell subsets. Representative flow cytometric illustration (MS case) of the in vitro effect of either minocycline or doxycycline supplemented at the highest concentration (50μg/ml) on apoptosis of sub-gated cell subpopulations of CD4 and CD8 T cells assessed by annexin V staining. Data are suggestive of lack of an in vitro effect of tetracyclines in inducting apoptosis and affecting viability. [file Image_4.jpeg]

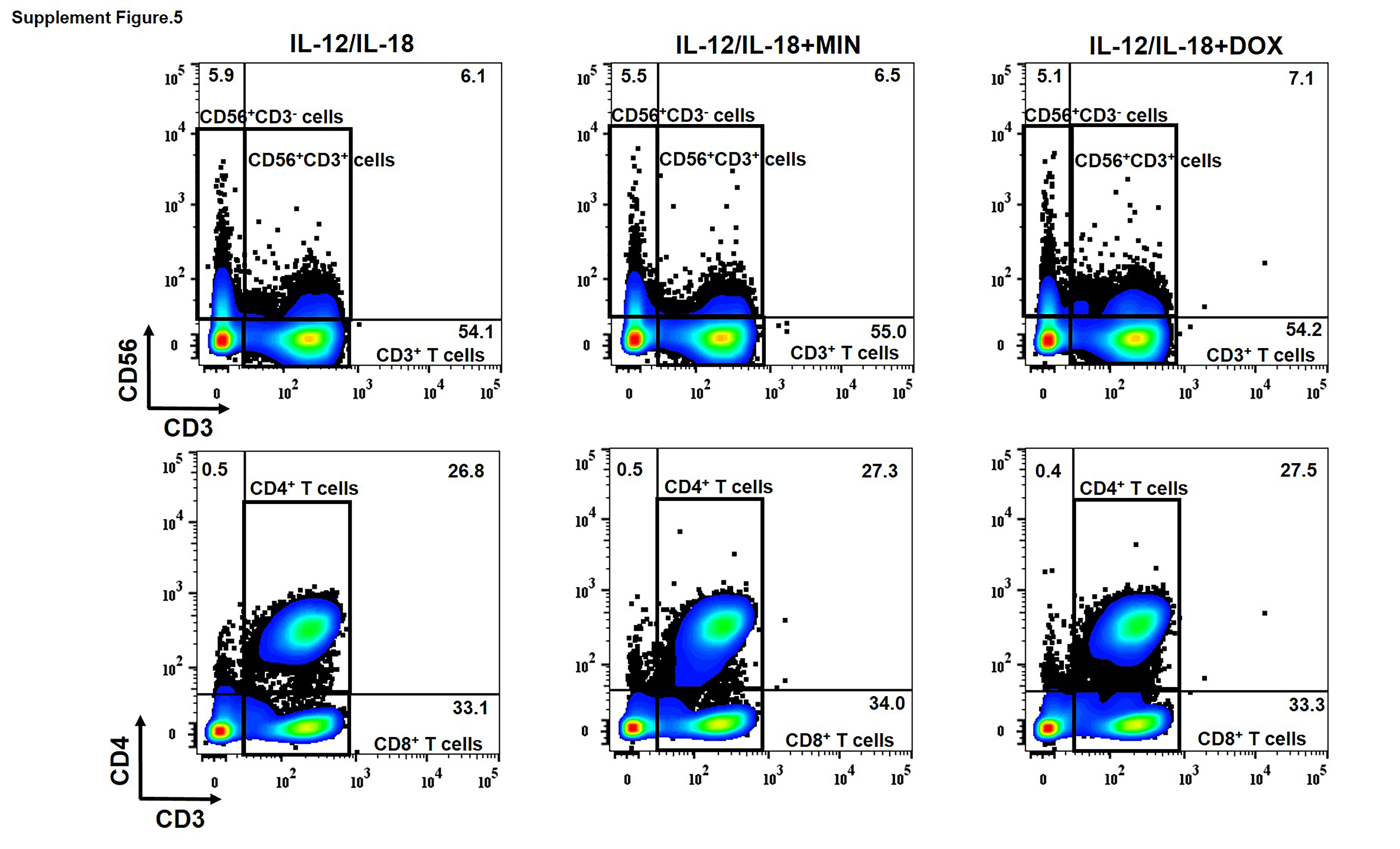

Supplement: Supplementary Figure 5 — Representative flow cytometric gating strategy of tetracycline treated IL-12 plus IL-18 stimulated PBMC subsets. Individual cell subsets from a representative MS case were sub-gated according to expression of CD3, CD4, CD8 and CD56 surface markers. All surface epitopes were sufficiently maintained and detected following cell activation with IL-12 plus IL-18 in the presence of minocycline and doxycycline. [file Image_5.jpeg]

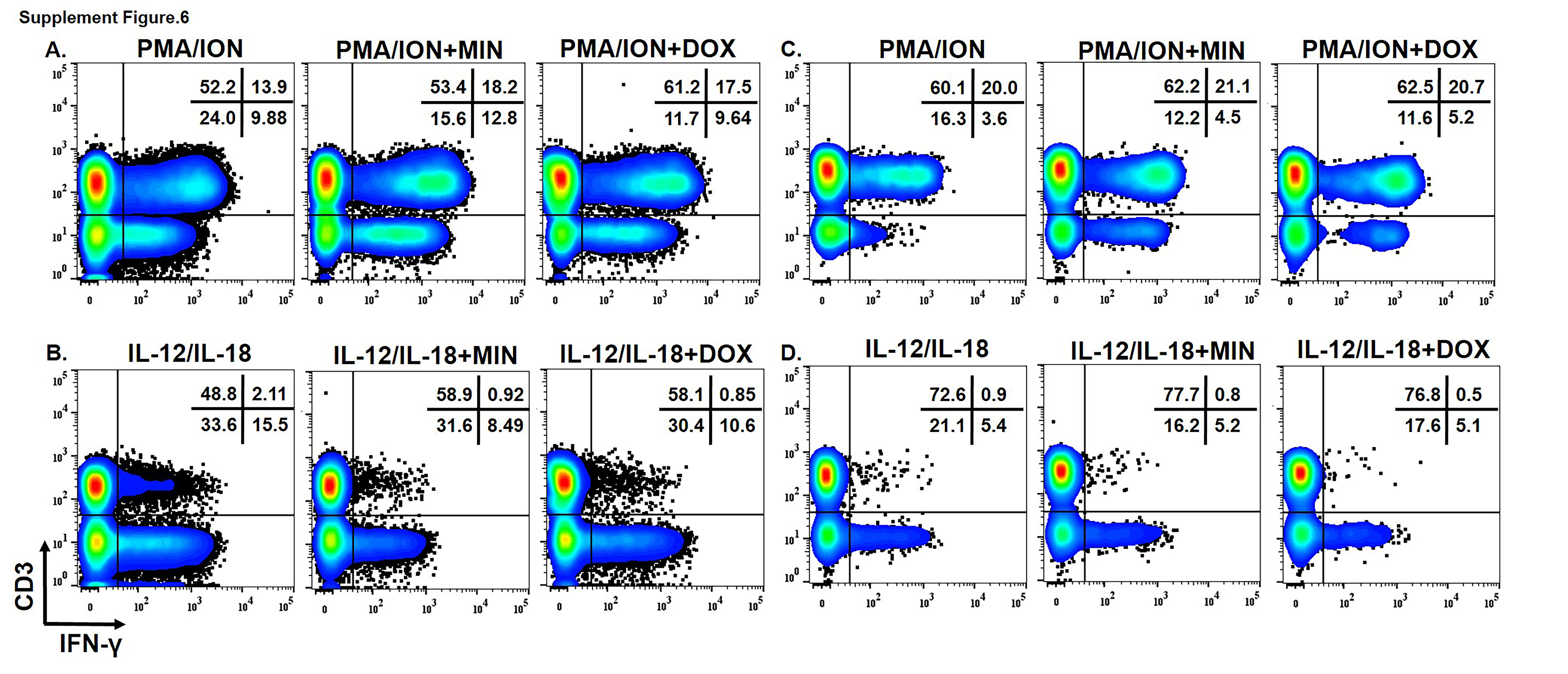

Supplement: Supplementary Figure 6 — Flow cytometric illustration of tetracycline-mediated effects on IFNγ production by PMA/ionomycin and IL-12 plus IL-18 stimulated CD3+ and non-CD3+ cell subsets from MS patients. Data of the effect of tetracyclines using different stimuli in a representative naïve MS case are shown in A and B and in a representative RRMS are shown in C and D. PBMCs were analyzed for IFN-γ production by flow cytometry following minocycline (MIN) or doxycycline (DOX) supplementation (50μg/ml) and simultaneous treatment with PMA plus ionomycin (A, C) or IL-12 plus IL-18 stimulation (B, D). CD3+ and non-CD3+ cell subsets were sub-gated according to expression of CD3 surface epitope. [file Image_6.jpeg]

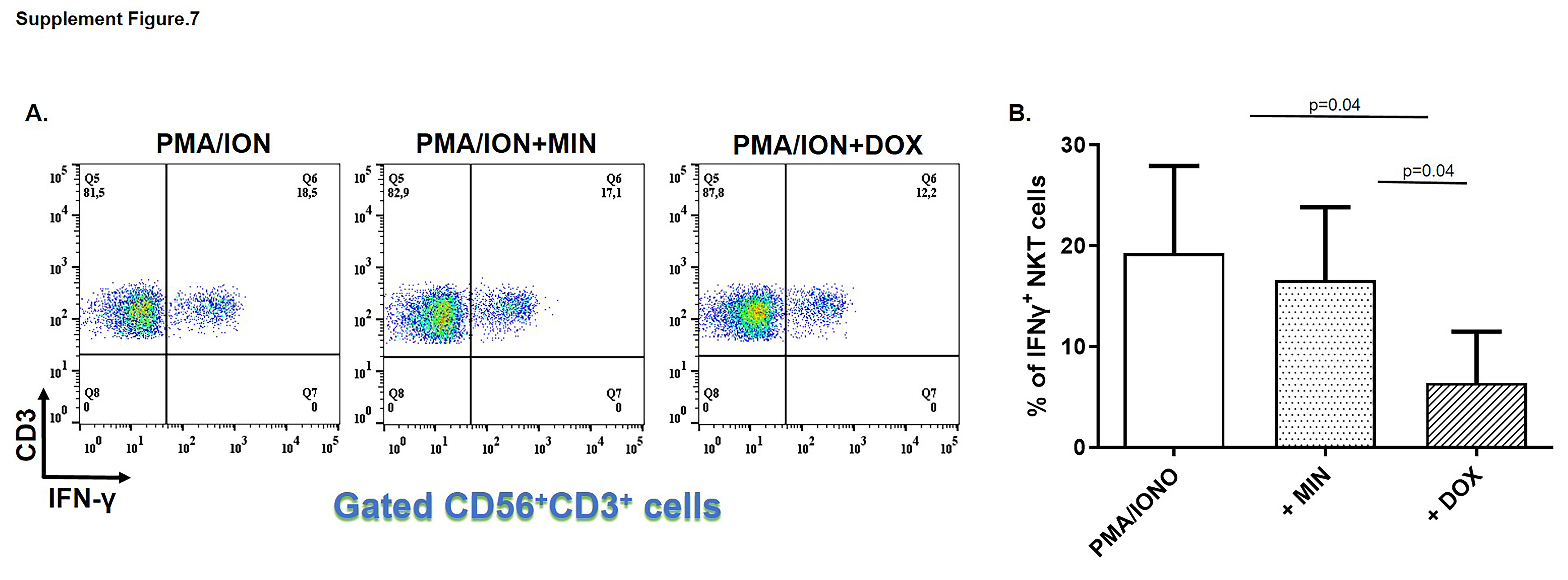

Supplement: Supplementary Figure 7 — Doxycycline-mediated inhibition of CD56+CD3+IFNγ+ T cells subsets following stimulation with PMA plus ionomycin. PBMCs from naïve MS patients (n=9) were analyzed for IFN-γ production by flow cytometry following minocycline (MIN) or doxycycline (DOX) supplementation and simultaneous PMA plus ionomycin stimulation (see also methods section). NKT cells were sub-gated according to expression of CD3 and CD56 surface markers. (A) CD3 versus IFN-γ Flow cytometry dot-plots in sub-gated NKT cells showing the frequency of IFN-γ+ NKTs following PMA/IONO, PMA/IONO/MIN and PMA/IONO/DOX treatment. (B) Box and whiskers graphical representation showing significant reduction in the percentages of IFN-γ-producing NKT cells in the presence of doxycycline in naïve MS. [file Image_7.jpeg]

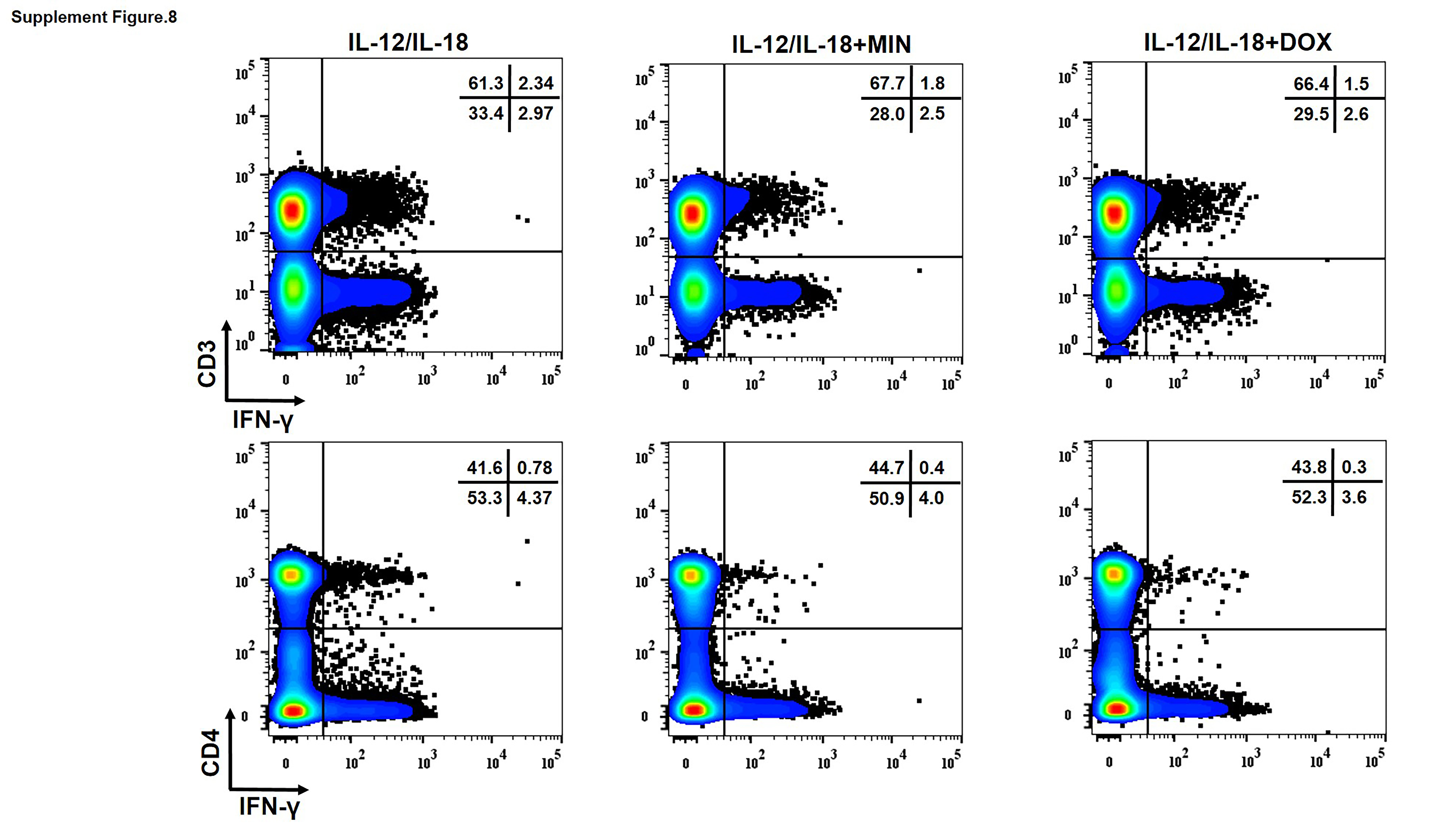

Supplement: Supplementary Figure 8 — CD4+IFN-γ+ cells represent T helper 1 (TH1) cells and not other CD4+ subsets like macrophage or DC subsets. PBMCs from MS patients were analyzed for IFN-γ production by flow cytometry following minocycline (MIN) or doxycycline (DOX) supplementation and IL-12 plus IL-18 stimulation (see also methods section).Th1 cells were assessed by staining against CD3, CD4 and intracellular IFN-γ simultaneously. A representative case of n=5 is illustrated where the effect of minocycline and doxycycline on IFN-γ production is clearly seen on CD3+ and CD4+ ie Th1 cells. [file Image_8.jpeg]
